# With equity in mind: Evaluating an interactive hybrid global surgery course for cross-site interdisciplinary learners

**DOI:** 10.1371/journal.pgph.0001778

**Published:** 2023-05-04

**Authors:** Barnabas Tobi Alayande, Zoe Hughes, Tamara N. Fitzgerald, Robert Riviello, Abebe Bekele, Henry E. Rice

**Affiliations:** 1 Center for Equity in Global Surgery, University of Global Health Equity, Kigali, Rwanda; 2 Program in Global Surgery and Social Change, Harvard Medical School, Boston, Massachusetts, United States of America; 3 Department of Global Health and Population, Harvard TH Chan School of Public Health, Boston, Massachusetts, United States of America; 4 Duke Global Health Institute, Duke University, Durham, North Carolina, United States of America; 5 Duke University School of Medicine, Duke University, Durham, North Carolina, United States of America; 6 Brigham and Women’s Hospital, Boston, Massachusetts, United States of America; Royal Infirmary of Edinburgh and University of Edinburgh, UNITED KINGDOM

## Abstract

There is limited understanding of the role of transcultural, cross-site educational partnerships for global surgery training between high- and low- or middle-income country (LMIC) institutions. We describe the development, delivery, and appraisal of a hybrid, synchronous, semester-long Global Surgical Care course by global health collaborators from widely different contexts, and evaluate the equity of the collaboration. The course was collaboratively modified by surgical educators and public health professionals with emphasis on collaboration ethics. Faculty from high-income and LMICs were paired to deliver lectures. To collaborate internationally, students and faculty participated either onsite or online. Perceptions and knowledge gained were quantitatively evaluated through participant and faculty cross-sectional surveys, using Likert scales, prioritization rankings, and free text responses analysed qualitatively. Equity was assessed using the Fair Trade Learning rubric and additional probes. Thirty-five learners from six institutions participated. Teams produced mock National, Surgical, Obstetric, and Anaesthesia Plans (NSOAPs) for selected LMICs, and reported a 9% to 65% increase in self-reported global health competencies following the course. Online learners had favourable perceptions of learning, but experienced connectivity challenges. Barriers to effective group work included time differences and logistics of communication for dispersed team members. Individuals taking the course for academic credit scored significantly higher than other learners in peer assessments of participation (8.56±1.53 versus 5.03±3.14; p<0.001). Using the Fair Trade Rubric, 60% of equity indicators were ideal, and no respondents perceived neo-colonialism in the partnership. Blended, synchronous, interdisciplinary global surgery courses based on “North-South” partnerships with a focus on equity in design and delivery are feasible but require careful and deliberate planning to minimize epistemic injustice. Such programs should address surgical systems strengthening, and not create dependency. Equity in such engagements should be evaluated and monitored in an ongoing fashion to stimulate discussion and continuous improvement.

## Introduction

Surgery has been increasingly recognized as key to enhancing global health, elevating pandemic preparedness, and achieving the Sustainable Development Goals [1, 2–6]. Large gaps exist in global surgical care, with the largest deficits in surgical infrastructure, human resources, financing, and education occurring in low- and middle-income countries (LMICs) [1]. These challenges have led to a surge of interest in global surgical education for students, researchers, clinicians, and educators [7–9].

Training of multidisciplinary learners from diverse countries, institutions, and socioeconomic backgrounds is key to improving equitable access and advocacy for global surgery [10]. In line with need for expertise in interdisciplinary tools to advance surgical care worldwide, creative education strategies are required to support a range of learners and future global health leaders [1, 7, 11]. There is limited understanding of the role of transcultural, multi-site educational partnerships for global surgery training in these skills between high-income country (HIC) and LMIC institutions. Even when these educational partnerships occur, partners must be careful that ethics of appropriate partnerships are maintained [12–16].

This paper describes the development of a hybrid, synchronous semester-long Global Surgical Care course between the University of Global Health Equity in Rwanda (UGHE) and Duke Global Health Institute (DGHI) targeted at multidisciplinary, cross-cultural learners from a variety of HIC and LMICs. We highlight key modifications to a historically in-person course to an international, blended cohort of remote and in-person learners [17]. In addition, we examine the ethics of collaboration and principles of equity between the partnering LMIC and HIC institutions, evaluate student cross-site group dynamics, and assess the educational outcomes and outputs of the program using the first and second Kirkpatrick framework [18].

## Methods

### Setting

The UGHE in Rwanda is a private, not-for-profit global health science university founded in 2015 as an initiative of Partners in Health [19]. Deliberately located in the rural Butaro community of Northern Rwanda, the institution aims to carry out health care training in the context of addressing the challenges in health equity needs across the region, utilizing an equity-focused, community-based education model. UGHE offers a flagship Masters in Global Health Delivery (MGHD), Bachelor’s Degree in Medicine and Surgery, and executive education on critical health system strengthening competencies [20]. The unique focus on equity and proximity to LMIC health systems aims to prepare learners to solve the most pressing contextual health challenges and promotes rural retention of trainees [21].

Founded in 2006, the DGHI seeks to achieve health equity for vulnerable individuals regionally and globally and supports global health activities across Duke University in partnership with collaborators around the world [22]. The focus of the institute is to prepare global health leaders through interdisciplinary education, to seek innovative solutions to the world’s most pressing global health challenges, and to partner for change by engaging international and local organizations. The DGHI offers several educational programs including a Master of Science in Global Health, an undergraduate co-major, and a Doctoral Scholars Program [22].

### Base course and curriculum

The course design was adapted from an existing semester-long graduate seminar in Global Surgical Care at the DGHI. Designed collaboratively by faculty in the Duke School of Medicine and the Duke Global Health Institute, the base course consisted of 12 topics built around the Lancet Commission on Global Surgery [17]. In-person class time was 2.5 hours weekly over 12–14 weeks, consisting of multiple learning methods. Students organized themselves into groups of 3–4 learners, with team-based research focussed on a LMIC of their choice for the length of the course. Weekly assignments fed into a final project—the development of a National Surgical Obstetrics and Anaesthesia Plan (NSOAP) for their chosen LMIC [17].

### Contextualization and review

In 2018, discussions began between members of UGHE and the DGHI to create a shared Global Surgical Care seminar. A formal strategic planning process was launched to ensure equity in course design, identification of shared goals, alignment with global health ethical standards for shared education programs, and contextualization to the needs of learners at all sites. The course design was carried out in a series of collaborative meetings over 6 months involving 7 UGHE and 5 DGHI participants (learners, educators, surgeons, and administrators). The contextualized course maintained the basic structure of team-based learning, flipped classroom approach, and final development of a NSOAP [17].

#### Frameworks

We used Glatthorn’s 1986 model framework for analysing the curriculum elements and guiding curriculum reform in 4 categories- curriculum structure, values, content, and process [23]. We also used Smith’s 1996 framework which guides curriculum developers to focus on curriculum as a body of knowledge to be transmitted, an attempt to achieve specific end-products, focus on the process, and praxis (S1 Fig) [23]. Finally, we used the Fair Trade Learning Rubric, which is a validated tool for assessing partnerships. It focuses on eight core principles including dual purposes, community voice and direction, commitment and sustainability, and transparency. In addition, it emphasizes environmental sustainability and footprint reduction, economic sustainability, deliberate diversity, intercultural contact and reflection, and global community building [24].

**Fig 1:**
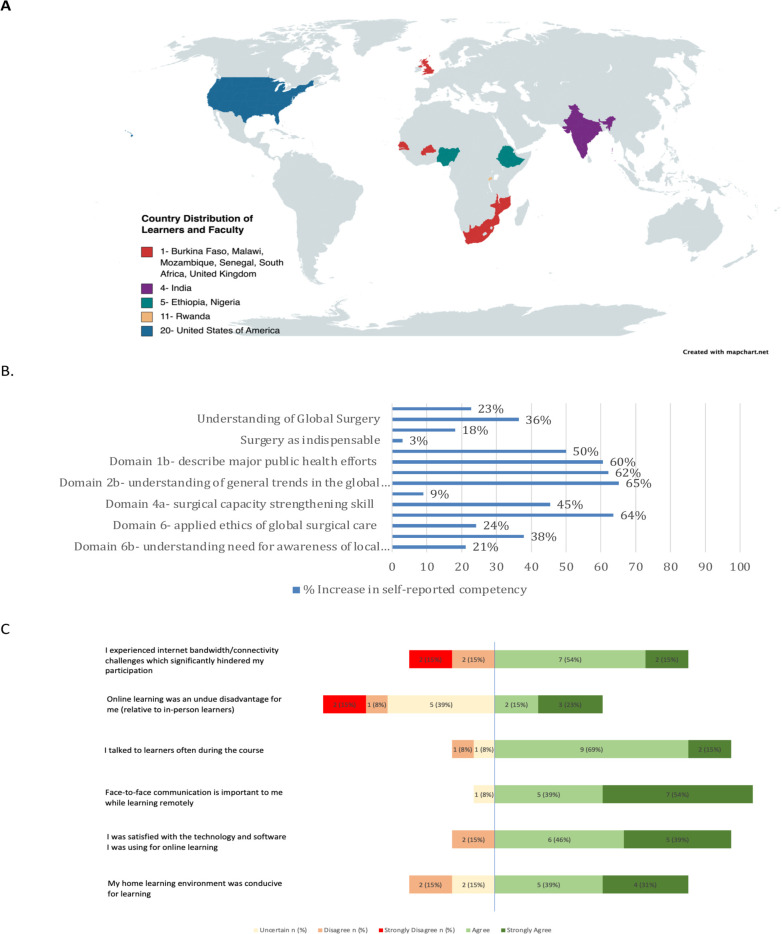
Location of learners, perceptions of online learning, and perceived increase in global surgery competencies. A. Physical location of learners and faculty for the 2021 course Created with mapchart.net. Direct link to base layer of the map at https://www.mapchart.net/world-subdivisions.html Republished from https://www.mapchart.net/ under a CC BY license, with permission from Minas Giannekas, original copyright 2023. B. Self-reported increase in Global Surgery competencies. C. Perceptions of online learning (n = 13).

Using a combination of Glatthorn and Smith’s models of curriculum theory and practice, the base curriculum was adapted to address the needs of a multi-disciplinary, international cohort of learners. This cohort consisted of remote and in-person learners from both LMICs and HICs (Fig 1) [23]. We considered the body of knowledge and content to be transmitted (number and theme of topics), in addition to the process and value models to be conveyed. This was in regard to assignments and assignment groups, diverse faculty selection, and twinning of LMIC and HIC faculty. We also focused the curriculum on NSOAPs as an end product. In addition, the team engaged in practical and technical deliberations about in-person versus remote attendance, internet challenges, availability of electricity, cultural differences, and institutional peculiarities [23].

#### Topic modifications

Five LMIC-based content experts affiliated with the UGHE reviewed the topics to ensure relevance to the African global surgery milieu. Material perceived as niche to North America by experts from Rwanda, Nigeria, and Ethiopia was flagged and adjusted. Content was reviewed to ensure that it was culturally relevant and contextual to African learners (Table 1).

**Table 1:** Course schedule, topics, and assignments.

Week	Topic	Student Assignments
1	Introduction: Lancet Commission on Global Surgery Key Messages	Students are divided into groups based on their region of interest and instructed to choose a country on which to focus for the remainder of the course
2*	Burden of Surgical Disease	Prepare an assessment of the global burden of disease for the focus country using the Global Burden of Disease Visualization Website [25]
3	Equity in Surgical Care	
4*	Infrastructure	Design a hospital-based survey to measure the infrastructure and current capacity
5	Innovation	
6*	Global Surgical Workforce	Prepare a report describing the current surgical workforce in the focus country. Design a study to validate this assessment and fill in the data that is missing.
7	Surgical Safety and Quality	
8*	Global Surgery Education	Design a surgical education program or initiative. Describe how you will evaluate the success of the program.
9	Microeconomics/ Financing	
10*	Macroeconomics	Provide a macro- and micro-economic profile of your focus country. Include national economic metrics, data on healthcare spending and financing and recommendations to improve financing of surgical care.
11	National Surgery Obstetrics and Anaesthesia Plan (NSOAP) Workshop	Prepare a NSOAP for the focus country. Describe how you will engage important stakeholders in the planning, approval and implementation of the NSOAP.
12, 13*	National Surgical Planning	NSOAP Presentations

*Shared UGHE/DGHI sessions.

#### Modification of mode of learning delivery

The course had historically been taught in-person at Duke University, while foundational courses for the first semester of UGHE’s MGHD are offered online. Many of the students enrolled in UGHE are from outside Rwanda and logistically cannot attend in person. For this collaboration, a hybrid blended approach was used with DGHI learners attending in-person and UGHE learners attending virtually via Zoom. The blended approach mitigated the risk and costs of travel, while still permitting students from different sites to participate.

At DGHI, the Global Surgery course qualifies as a ‘Research Methods’ course, and can be used to satisfy a Master’s degree requirement for instruction in research methods [17]. At UGHE, the Global Surgical Care Course is offered as an option for the ‘Principles in Global Health Equity’.

### Learner, faculty, and Learning Management System (LMS) selection

#### Learner selection

Selected students included Duke undergraduate, Masters and doctoral degree students, UGHE Masters students in Global Health Delivery, surgical residents from the College of Surgeons of East, Central, and Southern Africa from Mozambique and Malawi, and clinicians from the University of Rwanda, the West African College of Surgeons, Jos University Teaching Hospital, and Addis Ababa University. For LMIC learners, selection was premised on learner interest and carried out through the UGHE Masters program director in addition to Deans of respective institutions, and contacts made through UGHE’s partnerships. Attempts were made to balance the number of learners from DGHI with those from global sites to allow for cross-pollination of ideas among the group without domination of the group by a single group of learners.

#### Joint faculty selection and partnership in lecture delivery

Course faculty jointly selected experts in global surgery using a twinning model to minimize epistemic injustice [26]. Most topics were jointly covered by two faculty- one lecturer who lived, worked and was originally from a HIC and another who was resident, worked and was from a LMIC. These complementary views were aimed at generating a rich, balanced, and relatable conversation [27]. The team collaboratively assigned one core faculty to deliver structured didactics while the other shared related practical experiences. In the event of faculty from HIC residing and entrenched in LMICs, positionality statements would be used to appropriately determine where to place them, but we had no challenges in this regard.

#### Learning platform

The UGHE uses the Canvas platform as the student learning platform [28]. Duke University uses Sakai platform to manage student courses, interactions and grades [29]. Discussions on whether to utilize one or the other format, or to use a neutral, less automated platform informed the collective choice to use the Duke University platform for teaching and course content dissemination. To ensure that no institution imposed their academic standards on the other, pre-existing university learning management systems (Sakai and Canvas) were independently used for grading.

### Lecture time selection/logistics

Logistics for the timing of course delivery were collectively worked out by consensus. Sessions lasted two and a half hours from 8:30am EST/2:30pm CAT with the first hour dedicated to team presentation of assignments using flipped classroom pedagogy. Two teaching assistants (TAs) representing DGHI and UGHE were involved in administration of course logistics. A Zoom platform was set up for the classes and multiple sound capture devices were placed around the Duke classroom.

### Learner on-boarding and course delivery

Faculty and student onboarding to the learning platforms was designed as a highly supported process, with TAs assigned to accompany those that experienced technological challenges or were unfamiliar with this software.

Learners were grouped based upon their geographic region of interest, and then teams self-selected a LMIC to focus their studies. The number of HIC and LMIC academic and professional learners was proportionally balanced across all groups to maintain equity in representation, academic commitments, and field experience.

Delivery of the course was adjusted to maximize interactive, blended learning. Every two weeks, learners were assigned a section of the Lancet Commission on Global Surgery and other selected readings [1]. Weekly group assignments were carried out and presented in class as pre-recorded presentations to mitigate disruptions due to poor internet connectivity. The assignments were based on the topic for the week, and required demonstrated understanding of lectures, readings, and mastery of research methods. For the final project, students were required to develop and submit a written NSOAP for their adopted country and prepare a pre-recorded oral presentation.

TAs were available to mentor and support all learners through in-person and virtual office hours at times acceptable for learners in represented time zones.

### Assignment grading

Grading of student assignments and final projects was performed collectively, based upon a pre-set vetted rubric. However, scores were converted to final grades based on the policy of each institution. Institutional academic policies were respected with emphasis on institutional autonomy.

### Ethics approval and informed consent

Ethical approval was obtained from the UGHE Institutional Review Board (UGHE-IRB/2021/058), and informed consent was obtained from students on electronic forms. No review was required from Duke University since UGHE had designed and approved the course quality improvement surveys. Consent was informed. Teaching assistants explained the aims of the quality improvement study, and that participation was voluntary. The voluntary nature of the survey was also documented clearly on the consent page of each survey. Only individuals that consented could advance beyond the first page of the survey. As part of a quality improvement process, all consenting learners were eligible, and participated in anonymous course evaluations on Kirkpatrick levels 1 and 2 with assessment of perceptions and attitudes concerning the course [18] using a face-validated, pre- and post- course survey hosted on secure Google forms (Google, USA). No minors participated in the survey, and we did not obtain any consent from parents or guardians.

### Course evaluation

Likert scales, multiple choice, prioritization rankings, and free text responses were used for assessments. Self-reported improvement in the Consortium of Universities for Global Health (CUGH) Global Health competencies adapted to Global Surgery was assessed [30]. Perception of attainment of Global Surgery competencies were assessed at baseline and immediately following the course on the first Kirkpatrick level. Peer participation evaluations were performed within teams on a 10-point scale based on involvement with assignment teams, depth and quality of engagement, group meeting attendance and communication, contribution to project leadership, and timeliness in responses to queries and submission of assignments. Equity in partnership engagements was assessed using the Fair Trade Learning rubric and additional probes [24]. Anonymized data was stored on an encrypted platform (Google Drive), and analysed on password protected, encrypted systems to protect from unauthorized access. Quantitative data analysis using Chi squares, and percentages was performed on JASP software [31]. Free text responses were analysed using constant comparative methods by group open coding involving one investigator and a research assistant. Disagreements were resolved through discussion [32].

### Patient and public involvement

It was not appropriate to involve patients or the public in designing, conducting, or reporting this work.

### Financial disclosure

HR- The Duke University Office of Global Affairs financially supported the course (teaching assistant support, faculty support (Grand Rounds), and course administration) through HR. https://global.duke.edu/about.

The funders had no role in study design, data collection and analysis, decision to publish, or preparation of the manuscript.

## Results

### Sociodemographics

One third of participants (n = 11) were in-person DGHI students while the rest (n = 24) were virtual UGHE students or professional attendees (Table 2). There were more female than male participants. Physical location of learners and faculty during the course is shown in Fig 1. Multidisciplinary teams produced mock NSOAPs for Columbia, Kenya, Pakistan, Rwanda, and Vietnam.

**Table 2:** Sociodemographic and academic characteristics of 2021 course participants.

Characteristics	n (%)
**Learner’s Institution (n = 35)**	
University of Global Health Equity	15 (43%)
Duke University Global Health Institute (DGHI)	11 (31%)
College of Surgeons of Eastern, Central and Southern Africa	4 (11%)
University of Rwanda*	3 (9%)
West African College of Surgeons (fellow)	1 (3%)
Other (Recent medical graduate)	1 (3%)
**Sex (n = 33)**	
Male	15 (45%)
Female	17 (52%)
Not Specified	1 (3%)
**Mode of attendance (n = 35)**	
In-person	11 (31%)
Online	24 (69%)
**Credit status (n = 35)**	
Taken for credit	26 (74%)
Not for credit	9 (26%)
**Current academic pursuit (n = 33)**	
Masters in Global Health	22 (67%)
Masters in Bioethics	1 (3%)
Continuing Medical Education	7 (21%)
Masters in Bioethics and Science Policy	1 (3%)
PhD	2 (6%)
**Future (5 year) goals (n = 33)**	
Medical School	4 (12%)
Residency	6 (18%)
Program management for NGO	7 (21%)
Healthcare Consulting	1 (3%)
Health Policy and Governance	7 (21%)
Global Health Academic Research	6 (18%)
Non-Global Health Academic Research	1 (3%)
Undecided	1 (3%)

*An additional two full-time surgeons audited the course

### Participant global surgery competencies

There was a 9% to 65.2% self-reported increase in global health competencies with greatest gains in understanding of global availability of surgery, anaesthesia and obstetric care health workers, skill in situational analysis, design of surgery specific-health interventions, and attitudes of collaboration, partnering, and communication (Fig 1, S1 Table).

### Cross-cultural small group learning dynamics

**Fig 2:**
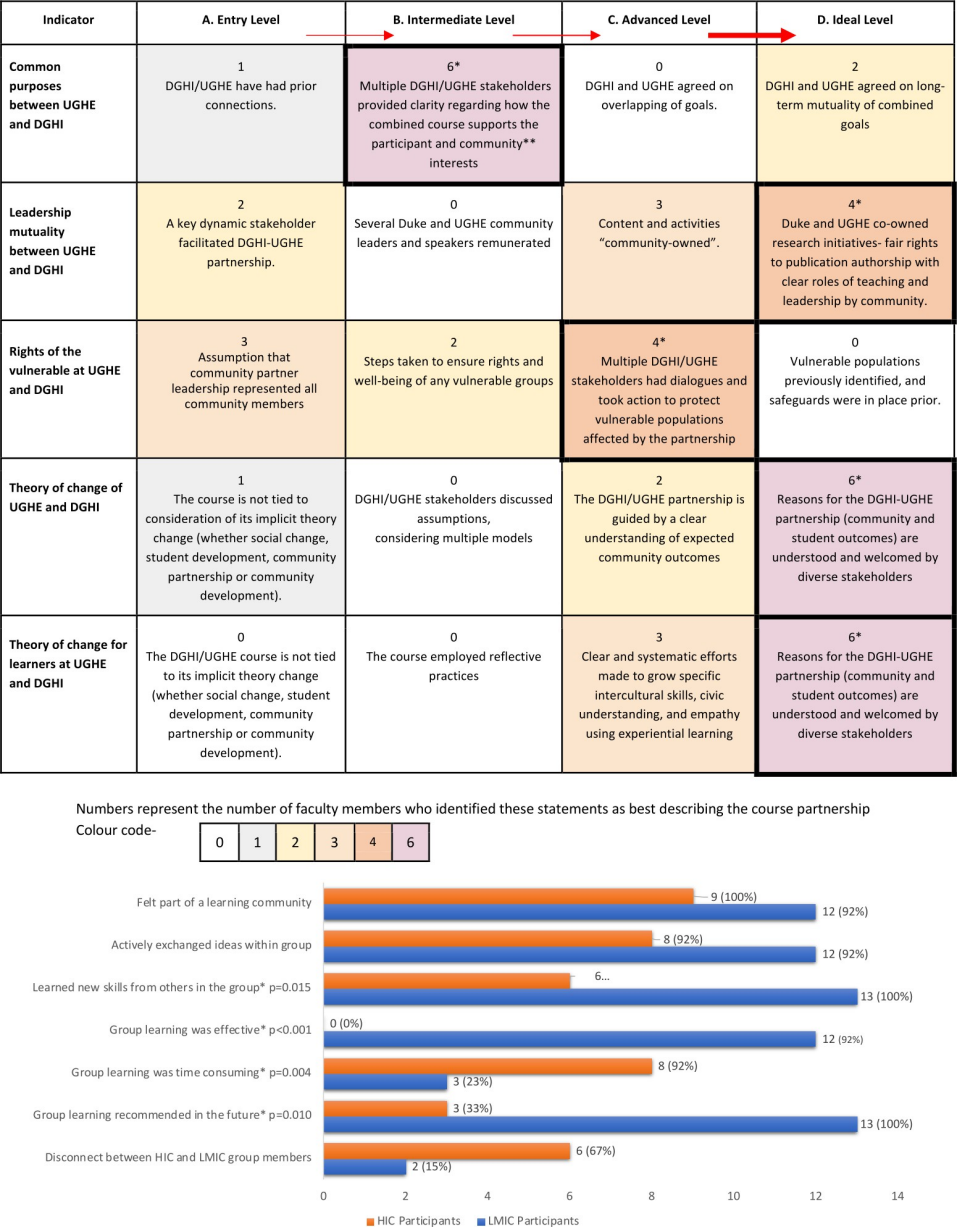
DGHI-UGHE course review using Fair Trade Learning rubric scoring and small group learning dynamics. *Modal level of agreement by faculty, fellows, and teaching assistants involved in organization and delivery of the course. **Community referred to the broader joint UGHE and DGHI communities.

Online learners (n = 13) had favourable perceptions of online learning but experienced challenges with internet connectivity during the course (Fig 1). Majority of learners preferred assignment teams of 3–5 persons (14; 64%). Interactive assignments were largely perceived as valuable, offering enhanced knowledge and cross-cultural thinking. Individuals taking the course for academic credit scored higher than professional learners in peer assessments of participation and engagement in small group assignments (8.56±1.53/10; 5.03±3.14/10; p<0.001) (S2 Table). The perceptions of small group dynamics varied significantly between HIC and LMIC learners, with HIC learners feeling that group learning was time consuming (p = 0.004) and not as effective (p<0.001) (Fig 2, S3 Table).

### Learning, course structure and support

A gender-balanced faculty was achieved with 7 female and 8 male facilitators teaching, in addition to 1 female (DGHI) and 1 male (UGHE) teaching assistant. Attendees generally felt supported through the course by TAs and faculty (n = 16; 73%). Of 18 hours designated as office hours for engagement with TAs, 7 hours (39%) were utilized by learners. They perceived that the course was logical and easy to follow (n = 19; 86%). All participants intended to apply what they had learned in research and practice, and 90% (20 out of 22) would recommend the course to others.

From free responses by 10 learners, 7 diverse themes were identified ranging from benefits of the course to barriers to effective group work (Table 3).

**Table 3:** Thematic analysis of additional learner observations.

Theme	Percent (%)	Low- and Middle-Income Country Student Free-Text Entries	High-Income Country Student Free-Text Entries
The course was beneficial and worthwhile	55.6	"This was an interesting course for me and sparked my interest to pursue a career in global surgery." LMIC student 15 "It was a very good course" LMIC student 22	“I really enjoyed the content in the course.” HIC student 16 “The idea was great.” HIC student 5 "I think that the collaborative nature of this class through working with students from UGHE has been a unique experience" HIC student 8
Time differences posed a challenge	44.4	"One major comment I would give is the timing and length of the class. For me personally it was during office hours plus considering the length was hard to fully pay attention and engage actively as I should have. Considering this for next time if the course can be considerate of the duration and the time zones of those involved would be great and more effective." LMIC student 15 "I will need all the recorded lectures to learn the course I missed especially on ethics and for reference to what was learned." LMIC student 22	"it was also quite challenging to organize the team for class deadlines on (US) Eastern Standard Time…it would maybe be easier for students for [to] form groups based on time zone or change due dates to account for time difference." HIC student 5 “Pairing them together created. . . time zone issues" HIC student 10
The challenge of unequal and inequitable contributions to group work	44.4	"(I suggest) equity in group assignment distribution" LMIC student 9	"the collaboration tended to operate more with Duke students taking on a larger amount of work." HIC student 5 "For my group especially a lot of the work was carried out by the Duke students since it was very difficult to motivate international students to have creative discussions on the direction of our assignments." HIC student 8 "Take some of the load off international students" HIC student 6
Concerns on differences in course grading priorities between HIC, LMIC students and non-graded learners	22.2		"but it (made) group work was made more difficult by having group members who had different grading/course policies and different priorities when we were assigned grades based on our entire group’s efforts for presentations as a whole." HIC student 8 "I think because grades mattered on my end and didn’t necessarily matter for them on theirs, there was a disconnect in motivation and a lack of want to put in effort." HIC student 10
Multiple logistics barriers to effective small group work in integrated LMIC/HIC assignment teams	22.2	Few things I detest…one of which is giving excuse. I was really pained yesterday…My batteries went flat. Two laptops, two mini, a tablet and a phone all went flat. We had no light (electricity) for about 24 hours. On restoration, it was ceased few minutes later (just as we were on the zoom call). I did all I could to gain 5 more minutes for my presentation, but all efforts proved abortive. I am really sorry. This is the second time I’ve been unable to present…just due to the place I found myself. LMIC student 2	"I don’t actually think it’s very conducive to learning due to a variety of factors such as bandwidth issues, meeting times, and in general getting work done. While I think working collaboratively like once or twice in the class is fine, it is logistically very taxing for large presentations and final reports." HIC student 8 "Pairing them [HIC, LMIC learners] together created. . . logistical problems, and differing intrinsic motivation." HIC student 10
HIC student dominated small group leadership	11.1		"the collaboration tended to operate more with Duke students taking on. . . more of a leadership role since it was assumed they have more experience which is not always the case" HIC student 5
Preference for smaller, more in-depth assignments	11.1		"Furthermore, I actually would have preferred more smaller assignments as I think I would have been able to delve deeper in certain topics than I could with just a 7 minute presentation shared among 7 people." HIC student 8

### Principles of equity between hosting LMIC and HIC institutions guiding curriculum design and course delivery

Surveys of HIC and LMIC faculty perceptions on the equity of the partnership showed no significant difference regardless of whether respondents were from HICs or LMICs (S4 Table). No neo-colonialism challenges were perceived during the course planning or execution. The Fair Trade Learning rubric was administered in a contextualized format to generate discussion (Fig 2). Out of five indicators, three (60%) were at ideal levels, one was advanced (20%), while the lowest scored indicator was at the intermediate level (20%).

## Discussion

### A case for creativity and equity in collaborative global surgery curriculum development and delivery

Global health curriculum, by definition, is not static, but responds to the interpretation and experiences of diverse participants in its reception and delivery [33]. Effective global health training collaborations between HIC and LMIC institutions often require adaptation of educational material from HICs to address realities of health care in resource challenged settings [34, 35]. We demonstrate that an evidence-based, collaborative academic global surgery course can be developed in an equitable manner which can optimize student experiences across diverse learners and institutions. It has been shown through similar HIC-LMIC education collaborations, that blended learning is a valuable approach to teach global health skills and enhance capacity for cross-national global health collaborations [33]. Our collaboration maximizes in-person and distance learning, and brings together LMIC and HIC learners in cross-cultural small groups, resulting in practical immersion of all learners into the dynamics of global health research, training, and academia, with first-hand experience of the advantages and the challenges of such partnerships.

Partnered global health (and indeed global surgery) courses should be intentionally crafted as ethical and mutually beneficial [12, 36, 37]. For this course, facilitators paid strict attention during course design and delivery that there were no elements of neo-colonialism, and that all course adaptations were based on the Fair Trade Learning rubric [12, 24]. Such collaborations, followed by audits of ethical structure, process, and outcomes, can contribute to equity in global surgery education and should be encouraged [36, 38]. As demonstrated, it is crucial that all partners are brought to the table from the beginning for meaningful and equitable partnership [39]. Global health equity has been recently defined as “mutually beneficial and power-balanced partnerships and processes leading to equitable human and environmental health products on a global scale” [40]. Power structures, and potential barriers to true partnerships need to be recognised and addressed [12, 36].

Many programs and courses in global health and global surgery are not accessible to many LMIC learners [41, 42]. A 2021 scoping review on Global Surgery Education and Training Programmes by O’Flynn *et al* failed to identify any academic global surgery specific education programmes in LMICs [43]. Apart from the fact that few academic global surgery institutions are located in LMICs [7, 43], the concept of “global surgery” is often misunderstood and the definition of this topic varies depending on context [44–47]. Despite the growing interest in academic global surgical care among learners from LMICs, the hurdles of acceptance into HIC institutions, tuition and transportation costs, and visas constitute often insurmountable barriers [48, 49]. Even after learning within an HIC context, questions of contextual applicability and the will of learners to return to their original contexts can be raised [50]. In addressing these challenges, this global surgical care course was envisioned to be an international partnership to support learners in their host countries and institutions, further ensuring training and retention of the next generation of global surgery leaders across various sites.

### Equity frameworks for global surgery education: Application of the Fair Trade Rubric

We ensured equity in the partnership throughout course design and delivery at institutional, faculty, and learner levels. We actively conducted processes to respect key components of social justice relative to equity in global health collaborations, including inclusive decision-making, group recognition and affirmation, promotion of collective well-being, ensuring self-development through capacity building, and avoidance of unequal power relations [14]. Collective course faculty selection, collaborative lecture delivery, joint selection of student groupings, and respect for institutional policies were a few ways that equity was pursued in the partnership. Available resources to help guide equity in international academic collaborations include the Fair Trade Learning Rubric, the Equity Tool for Valuing Global Health Partnerships [13, 51], the research fairness initiative tool, and the THET principles in partnership for Global Health framework among others [52, 53]. Evaluation of the course using the Fair Trade Learning rubric showed that mutuality of course leadership was ensured, and reasons for the partnership were understood and welcomed by diverse stakeholders, resulting in ideal institutional and learner theories of change. Using the Fair Trade Rubric, the least developed indicator was “the common purposes of the HIC/LMIC institutional partnership” which was rated at intermediate levels by most faculty. Faculty agreed that multiple stakeholders had provided clarity regarding how the combined course supports participant and community interests, but were yet to agree on long-term mutuality of goals for learners at all sites. In addressing this, since completion of the last course, both institutions have been working on a mutual, long-term, international training grant to support education and research capacity building at all sites.

### Multidisciplinary and interprofessional learning- course outputs and outcomes

The selection of a broad spectrum of learners ranging from undergraduate and graduate students (taking the course for academic credit), to practicing professionals (participating for continuing medical education) contributed a balance of academic rigor and practical insight obtained from real life experiences [33, 38, 54]. Deliberate twinning of global surgery educators from HICs and LMICs, with one faculty delivering academic background, tools, and frameworks and the other lecturer reinforcing this information using their practical, real-world, relatable experiences, is a novel model of delivery of academic global surgery training. The hybrid and interactive design of this course was necessary to bridge the gap between HIC and LMIC institutions, and to maximize learning using available expertise and educational resources [55]. This created a win-win situation for learners and faculty from both institutions, but was not without challenges of technology, logistics, time-zone differences, and heightened need for cultural competencies, similar to previous documented attempts [33, 56, 57].

Following the course, all students agreed that surgery is an indispensable part of healthcare. The impact of the course was seen in the increase in self-reported global surgery competencies across all domains, the desire of all participants to apply knowledge gained, and the high rate of course recommendation. The greatest gains in global surgery competencies (>60% increase) were in knowledge of the global burden of surgical disease, globalization of health and healthcare, strategic analysis, and collaboration, partnering and communication. This is in keeping with the core emphasis of the course on health system strengthening through the design of NSOAPs as a strategy to address the global surgical burden, and the collaborative small groups employed through the program [58]. These areas of gain were also strongly reflective of the two-weekly interactive student assignments and underscored their important contribution to learning. Learners highly valued these interactive assignments, as they enhanced knowledge and stimulated cross-cultural thinking. The greatest skill gains were in the domain of strategic analysis, and this also reflects the emphasis of the course.

### Small group dynamics and socio-cultural challenges

Complex logistics for team meetings, internet access, electricity, time management, leadership, and varied perceptions of workload were experienced by both HIC and LMIC learners. These challenges are common in global health, and as the course provides an early immersion into real-world cross-site partnerships [56, 59]. Global health has been described as a field full of commodities and diverse forms of capital [60]. Cultural capital (including owning the language of research, styles of speech and accent, and having credentials), social capital and networks, financial capital, and symbolic legitimacy are a few examples that individuals involved in global health must learn to recognize and utilize equitably in global health interactions [60]. Practical examples of shared capital include exchange of commodities like authorship, presentation and training opportunities, funding, and notoriety [60].

Small assignment groups were heterogeneous, consisting of HIC and LMIC partners, students and practicing professionals, and individuals with varying prior exposure to global health. Such multidisciplinary, interprofessional, and inter-institutional approaches have been found to be useful in delivery of global health courses [61]. This style enhances the learners’ ability to work in teams, to communicate effectively, and to provide improved patient-centred healthcare solutions [62–64]. In addition, interdisciplinary learners by necessity develop wider thinking, diversify their opportunities and interests, enhance critical thinking and holistic problem solving skills, experience unique peer mentorship, and learn more within a shorter space of time [54, 62, 65]. However, working in interdisciplinary teams is not without its drawbacks [66]. These include time pressures because of challenges in communication, the need for frequent collaboration for effectiveness, the tendency for incomplete decisions, the need for strong leadership, and increased possibility for disagreement- all of which played out in the student groups [67, 68].

#### Challenges within small groups: Differences in pose and gaze

Most learners agreed that small group interactive assignments were mutually beneficial, enhanced knowledge, and caused them to think more cross-culturally. Cross-cultural assignment groups made learners feel part of a community and encouraged active exchange of ideas. However, the perceptions of LMIC learners were divergent from HIC learners in several domains. In general, HIC learners did not agree that they learned new skills from others in the group (p = 0.015), saw group learning as ineffective (p<0.001), and that group learning was time consuming (p = 0.004), in contrast to LMIC students who strongly agreed to skills gain, and effective group learning in the small groups. Overall, learners from HICs were ambivalent about recommending the use of cross site small groups in future courses, while LMIC learners all strongly agreed or agreed that they should be used (p = 0.01).

Sociocultural and logistic challenges were largely responsible for these HIC driven concerns. Similar challenges have been described in a review of a similar collaboration between institutions in Uganda, Sweden and South Africa [33]. Time differences, for instance, posed a challenge to harmonized submission deadlines. Based on coordination theory, difficult communication, delay, challenges in clarification, and problems in rework are the cost for working in globally dispersed teams [69]. Students experienced considerable delay in their work and this reduced the efficiency of clarification of unclear aspects of joint assignment and increased the difficulty in reviewing work and making corrections. Potential solutions include prescribing mechanisms, means of communication, and times of meeting for the small groups. Research has shown that finding the appropriate mix of mechanisms that suits the collaborators’ needs for a task and collaborators preferences are more important than any particular coordination mechanism [69]. Flexibility in negotiating time windows is key [70]. Issues with internet connectivity, electricity access, and unfamiliarity with learning platforms were challenging for many learners.

The perceived disparity in mutual learning, where some HIC learners felt they did not learn new things from their LMIC colleagues was not an ideal outcome. Some HIC learners are unaware of the large amounts of bi-directional learning that can occur, and from an ingrained colonial perspective, may view global exchanges as unilateral [71]. More subtle issues including tendency to deference due to power dynamics [60], socio-cultural differences in taking initiative, spontaneous versus non-spontaneous leadership, perceptions of workload, intrinsic biases and assumptions were implied, but will require further, in depth qualitative study. HIC students felt they were doing more work in small groups because LMIC learners were not motivated to take on assignments while, in contrast, LMIC participants pointed at inequitable distribution of work. LMIC learners appeared less outspoken because of audio-visual challenges of their virtual engagement, and this might have easily been perceived as reduced interest in group leadership. This highlights the difference in prose and gaze from the divergent contexts. The team will consider conducting a strength, weakness, opportunity, and threat (SWOT) analysis and share results with the cross-cultural groups so that teams leverage on the strength of partnerships and learn from each other in a structured manner, independent of cultural or academic background. A quasi-experimental study on the effect of team imbalance on geographically dispersed teams compared the performance of completely co-located teams, teams with one isolated member, near-balanced teams, and evenly balanced teams. Interestingly, teams with one isolated member performed better than balanced or even wholly co-located teams in terms of handling coordination problems, identification with the team, and resolving conflict. This pattern created a novelty effect, without a perceived territorial threat, unlike balanced teams we had in this cohort, which gave rise to less identification with the entire team [72].

Peer assessments of small group engagement showed that lowest scores were received by individuals who were not taking the course for academic credit. While there is immense benefit in cross-fertilizing knowledge across professionals and students within groups, it must be recognized that motivations and expectations will differ. Professionals often have competing interests and, unlike students, may not be constrained by academic goals and need for competitive scores.

### Blended, hybrid, on-line learning and the pursuit of equity

Much improvement has occurred globally recently in the curation of online courses resulting from the COVID-19 pandemic [36, 73–75]. High quality online learning for limited resource settings is growing in volume and quality [76]. Of note, most of our online learners had a conducive home learning environment and were satisfied with the technology and software used. While co-located courses with online components are one step towards equity in access to global surgery education, online learning should not be considered as the ‘silver bullet’ for equity, as challenges exist with degree of participation, time zone differences, and the cost and quality of broadband [41, 57, 77]. Up to 70% of our learners experienced internet connectivity challenges. The United Nations Broadband commission has identified that over a billion people live in countries that fail to meet the recommendation for efficient internet use of “1 for 2”- where 1 gigabyte of data should not cost more than 2% of the average monthly income [77]. Even when cost is not a barrier to internet access, the quality of internet connection can hinder access [78]. Except for the challenge that UGHE learners are largely remote for the first semester of foundational courses, having in-person sessions for some topics with local faculty, with Zoom streaming to DGHI students could potentially reduce internet connectivity challenges and enhance the overall learner experience. The possibility of transferring the course to the early second semester, so that UGHE learners can be in-person, is being contemplated.

With the growth of academic global surgery collaborations [79], global surgical care seminars or webinars from the global north can pose similar challenges to those of short-term clinical surgical mission trips. These models are often not sustained, can create dependency, and fail to address longer-term surgical systems strengthening. HIC institutions should support the shift of the centre of gravity of global surgery education to areas of greatest need in low income settings [7]. The course is continuously being iteratively improved based on learner feedback, and will continue. In addition, this blended, interdisciplinary, inter-institutional course has served as one of the catalysts for a one year MGHD option in global surgery based in the LIC partner institution.

### Limitations

Although specific competencies for academic global surgery are under development, for now, no universally accepted set of competencies exist for academic global surgery [55]. Assessment of competencies was carried out with a broad adaptation of the CUGH global health competencies to global surgery [30]. Extensive reviews show that literature on global surgery competencies is largely by and for HIC institutions and trainees. A set of consensus global surgery competencies would have provided a more standardized benchmark for our assessment [55].

In addition, beyond perceptions and knowledge gained, higher Kirkpatrick levels (behavioural change and organizational performance resulting from the course) were not assessed [18]. Furthermore, improvement in competencies were self-assessed, introducing some subjectivity. Further evaluation of multidisciplinary small group dynamics is also essential, and this can be the focus of qualitative analysis of future courses.

## Conclusions

In conclusion, we present reflections on a blended, synchronous, interdisciplinary global surgery course with a focus on equity in course design and delivery. Our experiences through this education collaboration may help the design and implementation of similar educational partnerships and provide a framework for equitable engagement of learners and faculty. Multidisciplinary, collaborative small group assignments involving learners from HICs and LMICs can ensure that the benefits of cross-cultural global health collaborations are experienced by learners in a practical and mutually beneficial manner. It is our hope that our collaboration will provide a framework for other global surgery partners seeking to engage in effective, sustainable, respectful, reciprocal, organized, accountable, flexible, resourceful, and innovative courses across locations.

## Supporting information

S1 Checklist PLOS’ questionnaire on inclusivity in global research. (DOCX)

S1 Fig Curriculum theory and practice elements used to modify the course. (DOCX)

S1 Table Participant global surgery competencies at baseline and after the course. (DOCX)

S2 Table Peer assessments of participation and engagement in small group assignments. (DOCX)

S3 Table Small group learning dynamics. (DOCX)

S4 Table Faculty perceptions of equity in course development and delivery. (DOCX)

S1 Dataset Included data. (XLSX)
