# Supplementary material for: With equity in mind: Evaluating an interactive hybrid global surgery course for cross-site interdisciplinary learners
Source: PLOS Glob Public Health. 2023 May 4;3(5):e0001778. doi: 10.1371/journal.pgph.0001778 (PMC10159197; doi:10.1371/journal.pgph.0001778)
Supplement: S1 Checklist — (DOCX) [file pgph.0001778.s001.docx]

Inclusivity in global research

PLOS’ policy on inclusivity in global research aims to improve transparency in the reporting of research performed outside of researchers’ own country or community and ensures that PLOS publications reporting global research adhere to high standards for research ethics and authorship. Authors of relevant research articles may be asked to complete the questionnaire below, which outlines ethical, cultural, and scientific considerations specific to inclusivity in global research. This questionnaire may be requested when researchers have travelled to a different country to conduct research, if research uses samples collected in another country, research with Indigenous populations or their lands, or if research is on cultural artefacts. Researchers travelling to another country solely to use laboratory equipment will not normally be required to complete the questionnaire. However, the questionnaire can be requested at the journal’s discretion for any submission – if you have been requested to complete this questionnaire by the PLOS journal you submitted to, please do so.

Please complete the questionnaire below and include this as a Supporting Information file with your manuscript. Note that if your paper is accepted for publication, this checklist will be published with your article in the supporting information files. Please ensure that you reference the checklist in the main body of your manuscript. We suggest adding a subsection ‘Inclusivity in global research’ to your Methods section and adding the following sentence: “Additional information regarding the ethical, cultural, and scientific considerations specific to inclusivity in global research is included in the Supporting Information (SX Checklist)”

The questions have been designed to be applicable to a wide range of study types, and there are subsections for both human subjects research and non-human subjects research. If any of the questions are not relevant to your research please mark them as “N/A” as appropriate.

**Ethical considerations, permits and authorship**

*This section is applicable to all research types.*

Provide details as to who granted permissions and/or consent for the study to take place in the Methods section of your manuscript. This should include the names of **all** ethics boards, governmental organizations, community leaders or other bodies that provided approval for the study. If individuals provided approval refer to these people by their role or title but do not list their name(s).

Reported on page number: 12

If there were any deviations from the study protocol after approval was obtained please provide details of these changes in the Methods section of your manuscript.
Did this study involve local collaborators that are residents of the country where the research was conducted or members of the community studied? If you do not have any authors from said communities, please provide an explanation for this below.

Reported on page number: None.

Yes. This study was driven by Low Income Country collaborators and involved (actually was directed) by us. I, BA, as primary author and lead of the publication, alongside the senior author AB and Dean of the medical school, are resident in Rwanda and work in the country. This research was specifically designed by us local (Low-Income Country) partners and myself as junior course faculty, to assess and address equity in this international educational partnerships as part of our course assessment and quality improvement for future courses. This is a key priority of the University of Global Health Equity and its Centre for Equity in Global Surgery – the partnering institution from a low-income country. AB, a Low- and Middle- Income Country (LMIC) surgical educator and researcher who has extensive experience in leading and organizing international Global Health educational collaborations worked closely with HR, a High-Income Country (HIC) colleague and friend to conceptualize the course, and along with the entire local Rwanda based team, worked to adapt the course with a focus on equity for cross-site (HIC and LMIC) learners. AB provided oversight to the LMIC aspect of the partnership as the academic lead of the LMIC global health institution. A local Rwanda-based researcher, BA, drafted the evaluation protocol, and designed the study prior to larger team review as part of the institution’s quality improvement for UGHE courses. BA and AB also led the local application for ethics permissions. RR, though from a HIC (as a second-generation immigrant from an LMIC), works at the LMIC institution and provided support and mentorship, and reviewed drafts. They all contributed significantly to program design and reviewed the draft. There is a balance of HIC and LMIC researchers in this study authorship. All authors reviewed the final manuscript.

Everyone listed as an author should meet PLOS’ criteria for authorship and all individuals who meet these criteria should be included in the author byline, rather than the acknowledgements. Authorship criteria is based on the International Committee of Medical Journal Editors (ICMJE) Uniform Requirements for Manuscripts Submitted to Biomedical Journals - for further information please see here: <https://journals.plos.org/plosone/s/authorship>.

**Human subjects research (e.g. health research, medical research, cross-cultural psychology)**

Did you obtain written informed consent from a representative of the local community or region before the research took place? How did you establish who speaks for the community? Details of written informed consent obtained from study participants should be reported separately in the Methods section of your manuscript.

We obtained permission from the Ethics Review Committee that oversees the local community (University of Global Health Equity) UGHE-IRB/2021/058, and also had the approval of the Dean of the Medical School and Deputy Vice Chancellor (Academics and Research), who is a senior author for the research, which was aimed at quality improvement for iterative improvement of versions of the future course. We established that the UGHE Ethics Review Committee and the Deputy Vice Chancellor Academics and Research, and Dean of the Medical School (AB) spoke for the community- based on their community delegated authourity for oversight over research approvals and academics, and they were involved heavily in this manuscript.

How did members of the local community provide input on the aims of the research investigation, its methodology, and its anticipated outcome(s)?

The local community (Rwanda-based, UGHE) led the aims, designed the methodology without direction or influence from our Duke partners, and set the agenda. The ideas for this course assessment came from Rwanda/LMIC partners. Data was also collected, maintained and analysed by UGHE. The wider UGHE community provided input as we passed this through the ethics committee which includes several members of the local community.

When engaging with the local community, how did you ensure that the informed consent documents and other materials could be understood by local stakeholders?

All those that participated in this as respondents, had a tertiary education taught in English at the very least, and most were above Masters (in Global Helath Delivery), Masters in Public Health or surgical specialist level. All of them had English as their language of tertiary education, and were not disadvantaged by language in any way.

Teaching assistants (from the context) also explained clearly, both verbally and in writing, the informed consent, and option of filling out the surveys.

Will the findings of the research be made available in an understandable format to stakeholders in the community where the study was conducted (e.g. via a presentation, summary report, copies of publications, etc.)? Please provide details of how this will be achieved.

Yes, we have already had round table, in-person discussions with Rwanda UGHE students who have taken the course following course completion, alongside HIC and LMIC faculty in January, 2022. We discussed the results of this quality improvement study, pondered over course adjustments, and collaboratively received feedback on which we have based course adjustments and ammendments. We intend that this article will be published as open access, and UGHE community will have access to this freely and through the library. Our post-publication dissemination plan will include presentation at local and regional global health and global surgery conferences including CUGH. We hope to further engage with university leaders and leaders in global health, and surgical education who are involved in UGHE-Duke educational collaborations. The records will be made available to the local university libraries, to support iterations of future combined courses.

**Non-human subjects research using specimens/ animals collected as part of the study, or those housed in archival collections. Examples include archaeology, paleontology, botany and zoology.**

Did the permission you obtained from a local authority to perform the study include an agreement on access to outputs and benefit sharing? This may include procedures to enable fair distribution of the benefits and resources arising from the research performed. Please include any details of Prior Informed Consent and Benefit Sharing Agreements obtained. These may be required by field-specific regulations, for example the Convention on Biological Diversity (CBD) and the associated Nagoya Protocol.

Not applicable

If the material used in your study was imported, please A) provide the year it was imported and B) indicate whether permits were obtained to import/export the materials used, C) provide details of any permits obtained. If this information is not available, please indicate this.

Not applicable

If you used archival specimens, please state how the material used in your study was acquired by the institute it is held in and provide details of any permits obtained for the original excavations/ sample collection. If this information is not available, please indicate this.

Not applicable

How was the potential cultural significance of the materials collected in your study to local communities considered in your research design? Were Indigenous peoples and/or local researchers and institutions involved with archaeological excavations / collection of specimens? If so, please provide a description of their involvement.

Not applicable

If your manuscript includes photographs of human remains please indicate whether authors obtained permission from descendants or affiliated cultural communities to do so.

Not applicable
