# Supplementary material for: With equity in mind: Evaluating an interactive hybrid global surgery course for cross-site interdisciplinary learners
Source: PLOS Glob Public Health. 2023 May 4;3(5):e0001778. doi: 10.1371/journal.pgph.0001778 (PMC10159197; doi:10.1371/journal.pgph.0001778)
Supplement: S1 Fig — (DOCX) [file pgph.0001778.s002.docx]

**S1 Fig: Curriculum theory and practice elements used to modify the course(23)**

| **1.Smith’s model** of curriculum theory and practice | **Content** (syllabus) was co-developed/ modified. | The **Values and Process** were co-defined and agreed upon (collaborative lectures and 2-weekly small group assignments). | The **Product** was collaboratively agreed upon (capacity to produce a NSOAP using multiple global surgical care research methodologies). |
| --- | --- | --- | --- |
|  |  | **Praxis** was recognized by both HIC and LMIC partners (Awareness of the advantage and challenges of collaborative global surgical care). |  |
| **2. Glatthorn’s model** of curriculum theory and practice (parallel to Smith’s model) | The **Theoretical** Elements were agreed upon | The **Practical** Elements were agreed upon | The **Productive** Elements were agreed upon |
