# Supplementary material for: With equity in mind: Evaluating an interactive hybrid global surgery course for cross-site interdisciplinary learners
Source: PLOS Glob Public Health. 2023 May 4;3(5):e0001778. doi: 10.1371/journal.pgph.0001778 (PMC10159197; doi:10.1371/journal.pgph.0001778)
Supplement: S1 Table — (DOCX) [file pgph.0001778.s003.docx]

**S1 Table: Participant Global Surgery competencies at baseline and after the course**

|  | CUGH Domains | Self-reported Global Surgery competencies | Timing | Strongly Agree n (%) | Agree  n (%) | Uncertain n (%) | Disagree n (%) | Strongly Disagree n (%) | Percentage increase in self-reported competency (Agree and Strongly Agree) (%) |
| --- | --- | --- | --- | --- | --- | --- | --- | --- | --- |
| 1 |  | I have a good understanding of Global Health | Pre-course (N=33) | 2 (6.0) | 16 (48.5) | 8 (24.2) | 5 (15.2) | 2 (6.1) | 22.7 |
|  |  |  | Post-course (N=22) | 9 (40.9) | 8 (36.3) | 3 (13.6) | 2 (9.1) | 0 (0.0) |  |
| 2 |  | I have a good understanding of Global Surgery | Pre-course (N=33) | 1 (3.0) | 11 (33.3) | 10 (30.3) | 6 (18.2) | 5 (15.2) | 36.4 |
|  |  |  | Post-course (N=22) | 10 (45.5) | 6 (27.3) | 1 (4.5) | 4 (18.2) | 1 (4.5) |  |
| 3 |  | I understand the Global Health challenges faced in low resource contexts | Pre-course (N=33) | 7 (21.2) | 17 (51.5) | 4 (12.1) | 4 (12.1) | 1 (3.0) | 18.2 |
|  |  |  | Post-course (N=22) | 10 (45.5) | 10 (45.5) | 1 (4.5) | 1 (4.5) | 0 (0.0) |  |
| 4 |  | Surgery is an indispensable part of healthcare | Pre-course (N=33) | 23 (69.6) | 9 (27.3) | 1 (3.0) | 0 (0.0) | 0 (0.0) | 3.03 |
|  |  |  | Post-course (N=22) | 19 (86.3) | 3(3.6) | 0 (0.0) | 0 (0.0) | 0 (0.0) |  |
| 5 | Global Burden of Disease- Domain 1a (knowledge) | I can describe the major causes of surgical morbidity and mortality around the world, and how the risk of disease varies with regions | Pre-course (N=33) | 1 (3.0) | 8 (24.2) | 13 (39.4) | 8 (24.2) | 3 (9.1) | 50.0 |
|  |  |  | Post-course (N=22) | 6 (27.3) | 11 (50.0) | 3 (13.6) | 1 (4.5) | 1 (4.5) |  |
| 6 | Global Burden of Disease- Domain 1b (knowledge) | I can describe major public health efforts to reduce disparities in surgical aspects of global health | Pre-course (N=33) | 0 (0.0) | 7 (21.2) | 9 (27.3) | 14 (42.4) | 3 (9.1) | 60.6 |
|  |  |  | Post-course (N=22) | 8 (36.4) | 10 (45.5) | 2 (9.1) | 1 (4.5) | 1 (4.5) |  |
| 7 | Globalization of Health and Health Care- Domain 2 (knowledge) | I have a good understanding of how the globalization of health, health systems, and national surgery planning affect the delivery of timely, safe, and affordable surgical care | Pre-course (N=33) | 1 (3.0) | 7 (21.2) | 10 (30.3) | 12 (36.4) | 3 (9.1) | 62.1 |
|  |  |  | Post-course (N=22) | 8 (36.4) | 11 (50.0) | 0 (0.0) | 2 (9.1) | 1 (4.5) |  |
| 8 | Social and Environmental Determinants of Health- Domain 2b (knowledge) | I have a good understanding of the general trends and influences in the global availability and movement of surgery, anesthesia and obstetric care health workers | Pre-course (N=33) | 0 (0.0) | 7 (21.2) | 7 (21.2) | 15 (45.5) | 4 (12.1) | 65.2 |
|  |  |  | Post-course (N=22) | 6 (27.3) | 13 (59.1) | 0 (0.0) | 2 (9.1) | 1 (4.5) |  |
| 9 | Social and Environmental Determinants of Health- Domain 3b (knowledge) | I can list major social and economic determinants of health and their impacts on the access to and quality of health services and on differences in morbidity and mortality between and within countries | Pre-course (N=33) | 4 (12.1) | 17 (51.5) | 2 (6.0) | 8 (24.2) | 2 (6.1) | 9.091 |
|  |  |  | Post-course (N=22) | 12 (54.5) | 4 (18.2) | 3 (13.6) | 2 (9.1) | 1 (4.5) |  |
| 10 | Capacity strengthening- Domain 4a (Skill) | I have an understanding on how to collaborate with a host or partner surgical organization to assess the organization’s operational capacity | Pre-course (N=33) | 1 (3.0) | 5 (15.2) | 9 (27.3) | 15 (45.5) | 3 (9.1) | 45.454 |
|  |  |  | Post-course (N=22) | 3 (13.6) | 11 (50.0) | 5 (22.7) | 2 (9.1) | 1 (4.5) |  |
| 11 | Collaboration, Partnering, and Communication- Domain 5 (knowledge, skill and attitude) | I can describe successful collaboration between local, regional, and international entities that can improve surgical care | Pre-course (N=33) | 2 (6.1) | 4 (12.1) | 12 (36.4) | 10 (30.3) | 5 (15.2) | 63.636 |
|  |  |  | Post-course (N=22) | 4 (18.2) | 14 (63.6) | 1 (4.5) | 2 (9.1) | 1 (4.5) |  |
| 12 | Ethics- Domain 6, (knowledge, skill and attitude) | I can apply basic principles of ethics to global surgical care. | Pre-course (N=33) | 6 (18.2) | 13 (39.3) | 6 (18.2) | 7 (21.2) | 1 (3.1) | 24.2 |
|  |  |  | Post-course (N=22) | 11 (50.0) | 7 (31.8) | 2 (9.1) | 1 (4.5) | 1 (4.5) |  |
| 13 | Ethics- Domain 6a, (knowledge, skill and attitude) | I have a good understanding of and an ability to resolve common ethical issues and challenges that arise in working within diverse economic, political and cultural contexts as well as working with vulnerable populations and in low resource settings to address global health/ global surgery issues. | Pre-course (N=33) | 3 (9.1) | 10 (30.3) | 11 (33.3) | 7 (21.2) | 2 (6.1) | 37.9 |
|  |  |  | Post-course (N=22) | 3 (13.6) | 14 (63.6) | 2 (9.1) | 2 (9.1) | 1 (4.5) |  |
| 14 | Ethics- Domain 6b (knowledge) | I have a good understanding of the need to demonstrate an awareness of local and national codes of ethics relevant to one’s working environment | Pre-course (N=33) | 4 (12.1) | 16 (48.5) | 6 (18.2) | 3 (9.1) | 4 (12.1) | 21.2 |
|  |  |  | Post-course (N=22) | 5 (22.7) | 13 (59.1) | 1 (4.5) | 2 (9.1) | 1 (4.5) |  |
| 15 | Ethics- Domain 6c, (Knowledge and skill) | I can apply the fundamental principles of international standards for the protection of human subjects in diverse cultural settings | Pre-course (N=33) | 3 (9.1) | 12 (36.4) | 8 (24.2) | 4 (12.1) | 6 (18.2) | 27.3 |
|  |  |  | Post-course (N=22) | 5 (22.7) | 11 (50.0) | 2 (9.1) | 3 (13.6) | 1 (4.5) |  |
| 16 | Professional practice- Domain 7c, (Skills and attitude) | I have a good understanding of the need to demonstrate the ability to adapt clinical or discipline-specific skills and practice in a resource- constrained setting | Pre-course (N=33) | 0 (0.0) | 19 (57.6) | 7 (21.2) | 4 (12.1) | 3 (9.1) | 28.8 |
|  |  |  | Post-course (N=22) | 5 (22.7) | 14 (63.6) | 0 (0.0) | 2 (9.1) | 1 (4.5) |  |
| 17 | Health Equity and Social Justice- Domain 8a (Knowledge and skill) | I have a good understanding of how to apply social justice and human rights principles in addressing global surgery problems | Pre-course (N=33) | 2 (6.1) | 15 (45.5) | 5 (15.2) | 8 (24.2) | 3 (9.1) | 30.3 |
|  |  |  | Post-course (N=22) | 6 (27.3) | 12 (54.5) | 2 (9.1) | 1 (4.5) | 1 (4.5) |  |
| 18 | Health Equity and Social Justice- Domain 8e (Attitude) | I have a commitment to social responsibility around surgery | Pre-course (N=33) | 9 (27.3) | 14 (42.4) | 9 (27.3) | 0 (0.0) | 1 (3.0) | 25.758 |
|  |  |  | Post-course (N=22) | 11 (50.0) | 10 (45.5) | 0 (0.0) | 1 (4.5) | 0 (0.0) |  |
| 19 | Health Equity and Social Justice- Domain 8f (Knowledge) | I have a good understanding and awareness of the surgical health care workforce crisis in the developing world, the factors that contribute to this, and strategies to address this problem | Pre-course (N=33) | 4 (12.1) | 9 (27.3) | 13 (39.4) | 6 (18.2) | 1 (3.0) | 25.758 |
|  |  |  | Post-course (N=22) | 12 (54.5) | 8 (36.4) | 0 (0.0) | 1 (4.5) | 1 (4.5) |  |
| 20 | Program management- Domain 9a (Knowledge and skill) | I have a good understanding of how to plan, implement, and evaluate an evidence-based program | Pre-course (N=33) | 2 (6.1) | 9 (27.3) | 8 (24.2) | 11 (33.3) | 3 (9.1) | 53.029 |
|  |  |  | Post-course (N=22) | 7 (31.8) | 12 (54.5) | 1 (4.5) | 1 (4.5) | 1 (4.5) |  |
| 21 | Sociocultural and political awareness- Domain 10a (Knowledge) | I have a good understanding of the roles and relationships of the major entities influencing global health and development | Pre-course (N=33) | 2 (6.1) | 12 (36.4) | 10 (30.3) | 7 (21.2) | 2 (6.1) | 48.484 |
|  |  |  | Post-course (N=22) | 9 (40.9) | 11 (50.0) | 0 (0.0) | 1 (4.5) | 1 (4.5) |  |
| 22 | Strategic Analysis, Domain 11c (Skill) | I have a good understanding of how to conduct a situational analysis across a range of cultural, economic, surgical contexts | Pre-course (N=33) | 0 (0.0) | 7 (21.2) | 9 (27.3) | 13 (39.4) | 4 (12.1) | 65.151 |
|  |  |  | Post-course (N=22) | 7 (31.8) | 12 (54.5) | 0 (0.0) | 1 (4.5) | 1 (4.5) |  |
| 23 | Strategic Analysis, Domain 11d (Skill) | I have an understanding of how to design surgical specific-health interventions based upon situation analysis | Pre-course (N=33) | 1 (3.0) | 5 (15.2) | 10 (30.3) | 12 (36.4) | 5 (15.2) | 63.636 |
|  |  |  | Post-course (N=22) | 6 (27.3) | 12 (54.5) | 2 (9.1) | 1 (4.5) | 1 (4.5) |  |
