# Supplementary material for: With equity in mind: Evaluating an interactive hybrid global surgery course for cross-site interdisciplinary learners
Source: PLOS Glob Public Health. 2023 May 4;3(5):e0001778. doi: 10.1371/journal.pgph.0001778 (PMC10159197; doi:10.1371/journal.pgph.0001778)
Supplement: S2 Table — (DOCX) [file pgph.0001778.s004.docx]

**S2 Table: Peer assessments of participation and engagement in small group assignments**

| **Participant Characteristics** | **n (%)** | **Mean peer assessment score out of 10 ± standard deviation*** | **Range of scores** | **Mann Whitney U**** | **p-value** |
| --- | --- | --- | --- | --- | --- |
| Individuals taking the course for academic credit | 26 (74.29%) | 8.56±1.53 | 3.00-10.00 | 209.00 | <0.001 |
| Individuals not taking the course for academic credit | 9 (29.71%) | 5.03±3.14 | 1.00-8.80 |  |  |
| HIC participants*** | 11 (31.43) | 9.50±0.49 | 8.60-10.00 | 245.00 | <0.001 |
| All LMIC participants | 24 (68.57%) | 6.80±2.67 | 1.00-9.50 |  |  |
| LMIC participants taking the course for credit (LMIC n=24) | 15 (62.50%) | 7.87±1.67 | 3.00-9.50 | 112.00 | 0.008 |
| LMIC participants auditing/ not taking the course for credit (LMIC n=24) | 9 (37.50%) | 5.03±3.14 | 1.00-8.80 |  |  |

*Scores based on peer perceptions of depth and quality of engagement, group meeting attendance/WhatsApp communication, timeliness in responses to queries and submission of their part of an assignment, and contribution to project leadership within the assignment teams

**Shapiro Wilk test suggested a deviation from normality in scores of LMIC participants

***All HIC participants took the course for credit.
