# Supplementary material for: With equity in mind: Evaluating an interactive hybrid global surgery course for cross-site interdisciplinary learners
Source: PLOS Glob Public Health. 2023 May 4;3(5):e0001778. doi: 10.1371/journal.pgph.0001778 (PMC10159197; doi:10.1371/journal.pgph.0001778)
Supplement: S3 Table — (DOCX) [file pgph.0001778.s005.docx]

**S3 Table: Small group learning dynamics**

| Perceptions of assignment group dynamics | LMIC or HIC participants | Strongly Agree  n (%) | Agree  n (%) | Uncertain  n (%) | Disagree  n (%) | Strongly Disagree n (%) | Χ² | df | p-value |
| --- | --- | --- | --- | --- | --- | --- | --- | --- | --- |
| I felt I was a part of a learning community | LMIC (n=13) | 8 (61.5) | 4 (30.8) | 1 (7.7) | 0 (0) | 0 (0) | 4.851 | 2 | 0.088 |
|  | HIC (n=9) | 2 (22.2) | 7 (77.8) | 0 (0) | 0 (0) | 0 (0) |  |  |  |
| I actively exchanged ideas within my group | LMIC (n=13) | 5 (38.5) | 7 (53.8) | 1 (7.7) | 0 (0) | 0 (0) | 2.178 | 3 | 0.536 |
|  | HIC (n=9) | 3 (33.3) | 5 (55.6) | 0 (0) | 1 (11.1) | 0 (0) |  |  |  |
| I learned new skills from others in the group | LMIC (n=13) | 10 (76.9) | 3 (23.1) | 0 (0) | 0 (0) | 0 (0) | 10.483 | 3 | 0.015 |
|  | HIC (n=9) | 1 (11.1) | 5 (55.6) | 2 (22.2) | 0 (0) | 1 (11.1) |  |  |  |
| Group learning was effective | LMIC (n=13) | 5 (38.5) | 7 (53.8) | 1 (7.7) | 0 (0) | 0 (0) | 18.553 | 4 | < .001 |
|  | HIC (n=9) | 0 (0) | 0 (0) | 5 (55.6) | 2 (22.2) | 2 (22.2) |  |  |  |
| Group learning was time consuming | LMIC (n=13) | 0 (0) | 3 (23.1) | 5 (38.4) | 2 (15.4) | 3 (23.1) | 15.450 | 4 | 0.004 |
|  | HIC (n=9) | 7 (77.8) | 1 (11.1) | 1 (11.1) | 0 (0) | 0 (0) |  |  |  |
| Group learning is recommended in the future | LMIC (n=13) | 6 (46.2) | 7 (53.8) | 0 (0) | 0 (0) | 0 (0) | 13.313 | 4 | 0.010 |
|  | HIC (n=9) | 0 (0) | 3 (33.3) | 4 (44.4) | 1 (11.1) | 1 (11.1) |  |  |  |
| There was a disconnect between group members from High-Income and Low- and Middle-Income Countries within my group | LMIC (n=13) | 0 (0) | 2 (15.4) | 6 (46.2) | 1 (7.7) | 4 (30.8) | 8.112 | 4 | 0.088 |
|  | HIC (n=9) | 3 (33.3) | 3 (33.3) | 1 (11.1) | 1 (11.1) | 1 (11.1) |  |  |  |
