# Supplementary material for: With equity in mind: Evaluating an interactive hybrid global surgery course for cross-site interdisciplinary learners
Source: PLOS Glob Public Health. 2023 May 4;3(5):e0001778. doi: 10.1371/journal.pgph.0001778 (PMC10159197; doi:10.1371/journal.pgph.0001778)
Supplement: S4 Table — (DOCX) [file pgph.0001778.s006.docx]

**S4 Table: Faculty perceptions of equity in course development and delivery**

|  | LMIC facilitators  n (%) | HIC facilitators  n (%) | X^2^ | df | p-value |
| --- | --- | --- | --- | --- | --- |
| Perceived neo-colonialism during planning or running of the course |  |  |  |  |  |
| Yes | 0 (0) | 0 (0) | 2.250 | 1 | 0.134 |
| No | 6 (100) | 2 (66.7) |  |  |  |
| Do not know | 0 (0) | 1 (33.3) |  |  |  |
| Perceived that HIC actors defined the course priorities |  |  |  |  |  |
| Yes | 1 (16.7) | 0 (0) | 2.571 | 2 | 0.276 |
| No | 5 (83.3) | 2 (66.7) |  |  |  |
| Do not know | 0 (0) | 1 (33.3) |  |  |  |
| Perceived that HIC partners set the metrics for course success |  |  |  |  |  |
| Yes | 0 (0) | 0 (0) | 0 | 1 | 1 |
| No | 4 (66.7) | 2 (66.7) |  |  |  |
| Do not know | 2 (33.3) | 1 (33.3) |  |  |  |
| Felt that there was an imbalance in program leadership which disadvantaged HIC students |  |  |  |  |  |
| Yes | 0 (0) | 0 (0) | 0.321 | 1 | 0.571 |
| No | 5 (83.3) | 2 (66.7) |  |  |  |
| Do not know | 1 (16.7) | 1 (33.3) |  |  |  |
| Felt that there was an imbalance in program leadership which disadvantaged LMIC learners |  |  |  |  |  |
| Yes | 0 (0) | 0 (0) | 0.321 | 1 | 0.571 |
| No | 5 (83.3) | 2 (66.7) |  |  |  |
| Do not know | 1 (16.7) | 1 (33.3) |  |  |  |
